# Facile Synthesis of Te and Ag_2_Te Microrods for Light-Activated Bending-Responsive Photodetectors

**DOI:** 10.3390/nano15151156

**Published:** 2025-07-26

**Authors:** Hsueh-Shih Chen, Kapil Patidar, Pen-Ru Chen

**Affiliations:** 1 Department of Materials Science & Engineering, National Tsing Hua University, Hsinchu 30013, Taiwan; 2 College of Semiconductor Research, National Tsing Hua University, Hsinchu 30013, Taiwan; 3 Department of Chemical Engineering & Materials Science, Yuan Ze University, Taoyuan 32003, Taiwan

**Keywords:** metal telluride nanocrystals, silver telluride (Ag_2_Te) nanomaterials, hot-injection synthesis, micronrods, optoelectronic application

## Abstract

In this study, we report the synthesis of Te and Ag_2_Te micron-sized rods (MRs) via a controlled hot-injection-based quenching process, enabling the control of rod morphology and enhanced crystallinity. Structural analysis confirmed that the synthesized Te MRs exhibit a trigonal phase, growing along the (110) direction, while Ag_2_Te MRs undergo a phase transformation into a monoclinic structure upon Ag doping. A simple and scalable photodetector (PD) was fabricated by drop-casting Te and Ag_2_Te MRs onto PET plastic films, followed by the application of Ag paste electrodes. The PD demonstrated room-light-induced photocurrent responses, which increased significantly upon mechanical bending due to the formation of additional conductive pathways between MRs. The Ag_2_Te-based bending sensor exhibited a fivefold enhancement in photocurrent compared to its Te counterpart and maintained high stability over 1000 bending cycles. These results highlight the potential of Te and Ag_2_Te MRs for use in flexible and wearable motion-sensing technologies, offering a simple yet effective approach for integrating 1D telluride nanostructures into scalable optoelectronic applications.

## 1. Introduction

Nanomaterials have gained significant attention due to their unique structural, electrical, optical, and mechanical properties. These materials, ranging from zero-dimensional (0D) quantum dots to one-dimensional (1D) nanorods and nanowires, and two-dimensional (2D) nanosheets, have opened new avenues in energy storage, optoelectronics, and sensing technologies [1,2,3,4,5,6]. Among them, 1D nanostructures—such as nanowires, nanorods, nanotubes, and nanobelts—stand out due to their high aspect ratio, superior charge carrier mobility, and anisotropic properties, enabling efficient charge transport and photo-absorption [7]. These attributes make them highly promising candidates for flexible optoelectronics, electrochemical devices, thermoelectrics, sensors, and catalysis [8,9,10,11].

Transition metal telluride (TMT), as an important member of the transition metal chalcogenides (TMCs), has gained a lot of interest recently due to its structural diversity, varying valence states, and unique electronic states compared to other chalcogenides [12,13]. Tellurium (Te) nanostructures, as p-type semiconductors with narrow bandgaps, have shown great potential in the field of optoelectronics, and in biomedical and electrochemical applications [14,15]. The ability to precisely engineer the crystal phase and morphology of Te-based nanomaterials, such as Ag_2_Te, Se-Te, Bi-Te, Co-Te Cu-Te, and Cu-Sn-Te systems, further expands their potential in thermoelectric and other applications [16,17,18,19,20,21]. For instance, the unique crystal structure of Te enables efficient electron transport and defect engineering, boosting reactive oxygen species and glutathione depletion in TeSe nano-heterojunctions, thereby amplifying radiotherapy efficacy [18]. In particular, Ag_2_Te-based nanostructures have gained attention due to their stability, tunable electronic properties, high thermoelectric performance, and narrow bandgap (~0.1–0.35 eV), which make them promising candidates for optoelectronic and photodetection applications [13,17,22,23,24]. The development of flexible Ag_2_Te/nylon thermoelectric films and Ag_2_Te-based composite films with high power factors and excellent mechanical flexibility demonstrates their promise for wearable thermoelectric devices [22,23]. Yu et al. studied the Ostwald ripening of Ag_2_Te in PbTe, showing that annealing improves thermoelectric performance by transforming nanoprecipitates and introducing Ag-decorated dislocations for enhanced phonon scattering and electron transport [25].

In recent years, photodetectors have driven significant advancements in sensing technologies, with applications in security surveillance, biomedical imaging, and automation [7,15]. While traditional detectors rely on infrared or ultrasonic sensors, Te nanostructures have emerged as promising alternatives due to their superior optoelectronic properties [26,27]. Te nanowires and heterostructures enhance charge transfer, while flexible Te-based detectors offer mechanical robustness [20,28,29]. Additionally, mixed-dimensional Te heterostructures, such as Te/MoSe_2_ and TeSe alloys, provide tunable bandgaps and polarization-sensitive detection, further optimizing performance [21]. However, challenges remain in precisely controlling nanostructure morphology, ensuring uniform orientation, and minimizing synthesis-induced defects.

Several Te nanostructure synthesis approaches have been reported, including hydrothermal and solvothermal methods, chemical/physical vapor deposition (CVD/PVD), and electrochemical deposition [12,13,26]. However, achieving uniform orientation and controlled growth remains a critical challenge, as Te nanostructures often form random or misaligned morphologies due to their quasi-1D crystal structure [14]. PVD has shown potential in inducing unidirectional growth, but optimizing key parameters—such as substrate properties and annealing temperatures—remains essential for scalability [28]. Additionally, TMTs, including Ag_2_Te, generally struggle with particle agglomeration and volume fluctuations, leading to structural degradation and reduced device stability over prolonged cycling [21,25]. Addressing these issues requires precise control over synthesis conditions, precursor concentrations, and growth techniques to engineer stable nano- and micron-scale rods with enhanced durability for reliable, scalable Te-based light-sensitive motion detectors.

**Figure 1:**
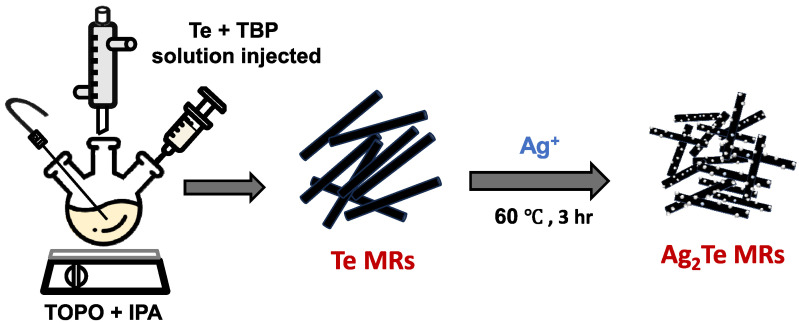
Schematic diagram of the hot-injection synthesis of Ag_2_Te MRs.

In this study, we report a simple wet-chemistry-based synthetic approach to Te and Ag_2_Te micron-sized rods (MRs) and demonstrate their application in light-sensitive bending and motion detection. A schematic diagram of the processes of Te and Ag_2_Te MR synthesis using the hot-injection method is shown in Figure 1. The photodetectors are fabricated by drop-casting the MRs onto a flexible plastic substrate, exhibiting a photocurrent response that varies with bending curvature. This behavior highlights the potential of Te and Ag_2_Te MRs for motion or deformation sensing. Our findings provide a scalable and effective strategy for incorporating these MRs into next-generation flexible sensing devices.

## 2. Materials and Methods

Chemicals: Isophthalic acid (IPA, 99%), trioctylphosphine oxide (TOPO, 90%), and tellurium powder (Te, 99.99%) were purchased from Sigma-Aldrich (St. Louis, MO, USA). Tri-n-butylphosphine (TBP) was obtained from Kanto chemical Co., Inc. (Tokyo, Japan). All reactants were used without further purification.

Synthesis of Te and Ag_2_Te MRs: Te MRs were prepared using a feasible hot-injection method [11,30,31]. In brief, 1.5 mmol Te powder was first dissolved in 3 mmol TBP by sonication to form a clear transparent solution at room temperature. A total of 20 mmol IPA and 10 mmol TOPO were added into a three-necked flask. The mixture was first heated to 150 °C, which was maintained for 10 min to remove undesired trace water, and then further heated to 320 °C. The Te stock solution was rapidly injected into the mixture. After reacting for 20 min, the mixture was naturally cooled to room temperature. The obtained Te MRs were separated by adding a solution of ethanol and toluene with centrifugation at 5000 rpm for 3 min. The supernatant was discarded, and the centrifugation process was repeated three times. The precipitated Te MRs were re-dispersed in ethanol for subsequent analyses.

Ag_2_Te MRs were prepared by doping Ag into the Te MRs. A total of 300 mg as-obtained Te MRs was added into 5 mL ethanol. Then, 10 mL 0.1 M AgNO_3_/ethanol solution was added into the Te MR solution. The mixture was stirred for 3 h at 60 °C. The product could be precipitated and re-dispersed in ethanol for the subsequent coating process.

Fabrication of light-sensitive motion detectors: A Te or Ag_2_Te bending sensor was fabricated by simply drop-casting the stock solution onto a polyester (PET) substrate. The casted film was approximately 10 mm in length and 3 mm in width. The film was dried in an oven in the air to evaporate the solvent. Then, a piece of 3M scotch tape (3M, St. Paul, MN, USA) was used to cover the central region (7 mm in length and 3 mm in width), leaving both sides, which were then coated with Ag paste as electrodes. The Ag electrodes were applied at both ends of the film, separated by approximately 7 mm, which corresponds to the length of the tape-covered region.

Characterization: Crystal structures were determined by X-Ray diffraction (Shimadzu XRD-6000, Shimadzu Corporation, Kyoto, Japan) using Cu K_α_ radiation. The XRD samples were prepared by dropping Te MRs or Ag_2_Te MRs onto a glass substrate. Scanning electron microscope (SEM, Hitachi SU8010, Hitachi High-Technologies Corporation, Tokyo, Japan) images were taken to examine the morphology of the nanomaterials and micromaterials. Transmission electron microscopy (TEM) images were taken using JEOL JEM-ARM200FTH (JEOL Ltd., Tokyo, Japan).

## 3. Results and Discussion

The synthesis of high-quality Te and Ag_2_Te MRs is essential for investigating their physical and chemical properties. Hydrothermal methods have been widely employed for synthesizing these structures due to their cost efficiency, versatility, and simplicity [24]. However, previous hydrothermal approaches have often resulted in nanostructures with unclear surface morphology and limited lateral dimensions, restricting their potential applications [13].

### 3.1. Growth of Te MRs

**Figure 2:**
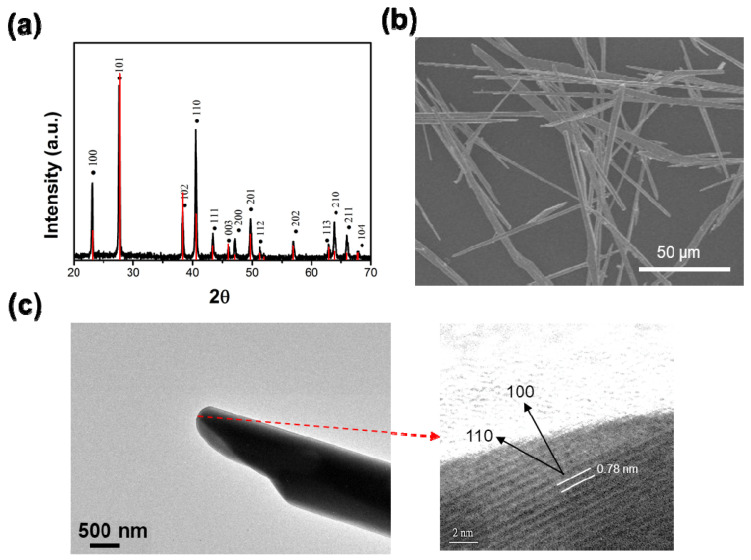
Characterization of the prepared Te MRs. (**a**) XRD pattern. The red line is from JCPDS #361452. (**b**) SEM image. (**c**) TEM image of a selected Te MR. The right inset shows a high-resolution image of the Te lattice fringe.

The present synthetic approach is based on the hot-injection-based quenching process, where nanocrystal growth occurs at a much faster rate. It has been found that both isophthalic acid (IPA) and trioctylphosphine oxide (TOPO) are essential for the formation of 1D nanostructures [30,32]. X-Ray diffraction (XRD) analysis confirmed that the synthesized Te MRs exhibit a hexagonal Te crystal structure, with all diffraction peaks matching the hexagonal phase of Te on the standard JCPDS #36-4152 card (Figure 2a). Although the most intense peak corresponds to the (101) plane, high-resolution TEM imaging at the tip of an individual Te MR (Figure 2c) confirms that the rods grow predominantly along the (110) direction. The scanning electron microscopy (SEM) image (Figure 2b) reveals that the Te MRs synthesized in a mixture of IPA and TOPO form long, micron-sized rods with widths ranging from 4 to 6 μm and lengths between 60 and 200 μm.

### 3.2. Growth of Ag_2_Te Rods

In this study, Ag_2_Te MRs were obtained by converting Te MRs through Ag^+^ doping in ethanol, forming a thermodynamically stable phase at room temperature (monoclinic Ag_2_Te). Typically, it can be prepared from the heating of solid silver and tellurium above 200 °C via a reaction equation:

(1) $$2Ag\left(s\right)+Te\left(s\right)\to {Ag}_{2}Te\left(s\right)$$

**Figure 3:**
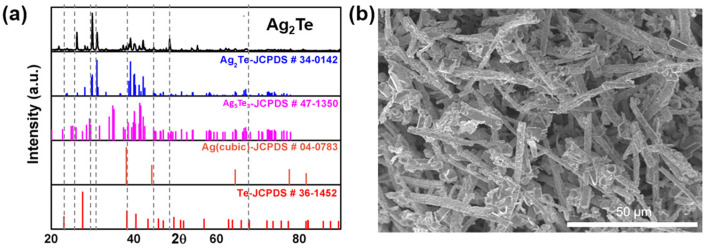
Characterization of Ag_2_Te MRs. (**a**) XRD pattern of Ag_2_Te MRs. (**b**) SEM image of Ag_2_Te MRs and related JCPDS cards for reference.

In the current study, Ag_2_Te MRs were directly converted from Te MRs by introducing Ag^+^ ions in ethanol at 60 °C for 3 h. The XRD analysis confirms the formation of Ag_2_Te MRs with a monoclinic structure, indicating that Ag doping successfully transforms Te into Ag_2_Te (Figure 3a). The morphology of Ag_2_Te MRs remains similar to that of Te MRs, as shown in the SEM image (Figure 3b). The Ag_2_Te MRs exhibit an average width of 2.2 μm and a length of 21.3 μm, significantly smaller than the original Te MRs. This size reduction could be attributed to structural changes and/or partial dissolution during the Ag doping process.

### 3.3. Light-Driven Bending Sensor

**Figure 4:**
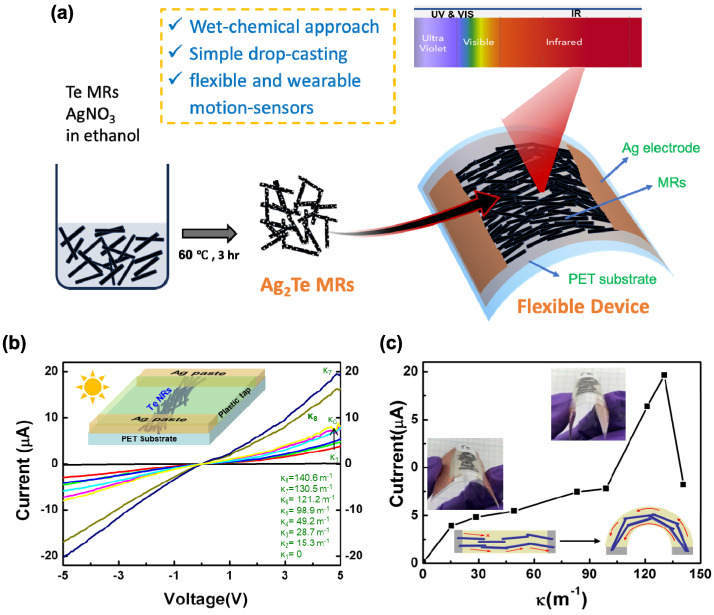
(**a**) Schematic illustration of the fabrication process for photosensitive motion detectors: Te MRs were drop-cast onto a PET film, covered with 3M scotch tape, and placed in contact with with Ag paste electrodes. (**b**) The photocurrent response at different bending curvatures under room lighting (~250 cd/m^2^). The inset shows the device with Ag paste electrodes spaced ~7 mm apart on a PET substrate. (**c**) Photocurrent variation as a function of bending curvature under ambient room lighting (~250 cd/m^2^) at a bias voltage of 5 V. The inset images show photographs of the sensors (**up**) and a schematic illustration (**bottom**) of photocurrent behavior in normal and bent conditions.

Both crystalline Te and Ag_2_Te MRs exhibit photosensitivity, making them suitable for photodetection applications. As shown in Figure 4a, a simple photodetector (PD) was fabricated by drop-casting Te MRs onto a PET plastic film under ambient conditions, eliminating the need for complex fabrication processes. To secure the MRs onto the substrate, a piece of standard 3M scotch tape was placed over them, followed by the application of Ag paste electrodes on both sides, as shown by the inset image in Figure 4b. The fabricated device exhibits a photocurrent of less than 100 pA under ambient room light. Interestingly, the photocurrent increases when the substrate is bent. This response can be systematically tuned by varying the bending curvature from 0 m^−1^ (κ1) to 130.5 m^−1^ (κ7), as shown in Figure 4b. This bending motion detector demonstrates an approximately linear relationship between 15 and 100 m^−1^, as depicted in Figure 4c. The increase in current is attributed to the formation of additional conductive paths between interconnected MRs, as the plastic substrate and cover film undergo mechanical strain, bringing the interlaced MRs into closer contact. The conducting mechanism of the Te MR is similar to that of the Ag nanowire (NW)-based transparent conductor film, where overlapping Ag NWs form interconnected pathways after post-annealing [33,34]. However, in the present case, Te MRs do not merge at the overlapping points but are instead held in place by the adhesive resin of the plastic tape. The enhancement of photocurrent upon bending primarily results from the mechanical compression of the substrate, reducing the spacing between adjacent MRs and thus significantly increasing the inter-rod contact area and connectivity. This enhanced connectivity notably decreases interfacial contact resistance, facilitating more efficient charge carrier transport. Additionally, mechanical bending may induce localized strain at the inter-rod contacts, altering the local electronic structure and potentially enhancing the separation and transport of photogenerated charge carriers. These combined effects contribute significantly to the overall improvement in photocurrent. Initially, the photocurrent remains low due to the limited contact points and conducting pathways between Te MRs. As the device bends, additional conductive paths form, leading to an increase in photocurrent (Figure 4c inset and Figure S1). However, when the curvature exceeds 131 m^−1^, the photocurrent drops abruptly due to structural damage in the MRs, leading to electrical breakdown.

**Figure 5:**
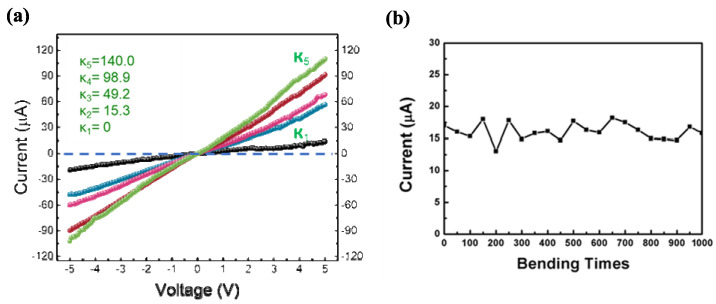
Photocurrent of the Ag_2_Te sensor caused by bending under room lighting (~250 cd/m^2^). (**a**) Photocurrent variation in the bending sensor fabricated using Ag_2_Te MRs under different applied voltages. Ag_2_Te MRs were prepared by introducing an AgNO_3_/ethanol solution into the as-prepared Te MR solution (κ_1_ (black), κ_2_ (blue), κ_3_ (pink), κ_4_ (red), and κ_5_ (green)). (**b**) Photocurrent stability of the sensor measured under room light after 1000 bending cycles at a curvature of κ_3_ (bias = 3V).

A similar motion sensor is fabricated using Ag_2_Te MRs, as shown in Figure 5a. The photocurrent of the Ag_2_Te MR sensor increases more than fivefold (under 5 V) compared to that of the Te MR sensor when the film is bent at κ5. The improved photocurrent could be caused by the relatively higher carrier mobility of Ag_2_Te (~6000 cm^2^/V·s) compared with Te (~900 cm^2^/V·s) [35]. The enhanced photocurrent observed in Ag_2_Te MRs may be influenced by the materials’ specific electronic properties. Although reports in the literature suggest higher carrier mobility for Ag_2_Te compared to Te, we acknowledge that our Ag_2_Te MRs are likely polycrystalline, which could reduce their effective mobility. Additionally, recombination behavior is also linked to material quality, and Te MRs may exhibit lower recombination rates than polycrystalline Ag_2_Te [36]. The increase in photocurrent with the curvature would follow the same mechanism as Te MRs, where additional conductive paths form between interconnected rods. The Ag_2_Te bending motion sensor also demonstrated excellent stability, with a photocurrent between 12 and 20 µA after 1000 bending cycles at a curvature of κ_3_ = 49.2 m^−1^, as shown in Figure 5b. This may be attributed to their shorter length and lower aspect ratio, which reduce bending-induced stress. Additionally, the polycrystalline nature of Ag_2_Te may allow slight grain-boundary slip, enhancing mechanical resilience during deformation. The photocurrent caused by bending the sensor works well under room lighting (~250 cd/m^2^). In a dark environment, the bending sensor does not operate, so light may function as a switch to activate the sensor and could enhance operational safety for future smart devices. The minimum activation light intensity depends on the loading and stacking morphology of the rods, and is currently under investigation. These light-driven bending detectors exhibit high feasibility for motion detectors and light-switching applications, particularly for detecting mechanical deformation. Their flexible, scalable nature makes them promising candidates for wearable motion sensors, security surveillance, and interactive optoelectronic systems.

## 4. Conclusions

In summary, we report a simple synthetic approach to prepare Te micron rods with high crystallinity. Ag_2_Te micron rods can also be prepared by reacting Te MRs with AgNO_3_ at 60 °C for 3 h. The light-switchable bending/motion detectors, prepared by a simple drop-casting process on a plastic substrate, exhibiting a photocurrent response under ambient light, which increased with bending curvature due to enhanced inter-rod contact. The Ag_2_Te-based motion sensor showed a fivefold enhancement in photocurrent in relation to Te and remained stable over 1000 bending cycles. These results underscore the feasibility of 1D telluride-based nanostructures for next-generation motion sensors, providing a promising route toward the development of flexible and high-performance optoelectronic devices for motion or deformation detection applications.

## Data Availability

The original contributions presented in this study are included in the article/Supplementary Materials. Further inquiries can be directed to the corresponding author.

## Supplementary Materials

The following supporting information can be downloaded at: https://www.mdpi.com/article/10.3390/nano15151156/s1, Figure S1: An illustration and a photograph of the Ag_2_Te bending sensor.
