# Supplementary material for: Facile Synthesis of Te and Ag2Te Microrods for Light-Activated Bending-Responsive Photodetectors
Source: Nanomaterials (Basel). 2025 Jul 26;15(15):1156. doi: 10.3390/nano15151156 (PMC12348639; doi:10.3390/nano15151156)
Supplement: Supplementary file 1 [file nanomaterials-15-01156-s001.zip › nanomaterials-3735132-supplementary.pdf]

Supporting Information

Facile Synthesis of Te and Ag_2Te Microrods for Light-Activated Bending-Responsive Photodetectors

Hsueh-Shih Chen [1,2,3,*], Kapil Patidar [1] and Pen-Ru Chen [1]

[1] Department of Materials Science & Engineering, National Tsing Hua University, Hsinchu 30013, Taiwan
[2] College of Semiconductor Research, National Tsing Hua University, Hsinchu 30013, Taiwan
[3] Department of Chemical Engineering & Materials Science, Yuan Ze University, Taoyuan 32003, Taiwan
* Correspondence: chenhs@mx.nthu.edu.tw

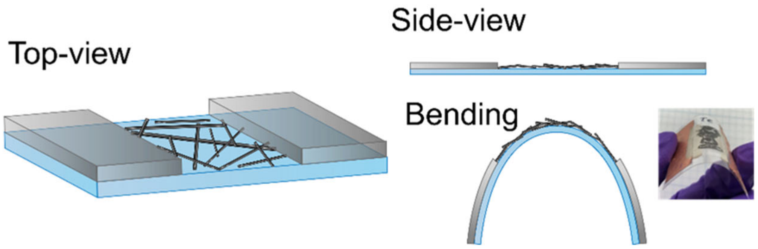

Figure S1. Illustration and a photograph of the Ag_2Te bending sensor.
